# SGC Tests for Influence of Material Composition on Compaction Characteristic of Asphalt Mixtures

**DOI:** 10.1155/2013/735640

**Published:** 2013-06-02

**Authors:** Qun Chen, Yuzhi Li

**Affiliations:** ^1^School of Traffic and Transportation Engineering, Central South University, Railway Campus, Changsha 410075, China; ^2^Changsha University of Science and Technology, Changsha 410076, China

## Abstract

Compaction characteristic of the surface layer asphalt mixture (13-type gradation mixture) was studied using Superpave gyratory compactor (SGC) simulative compaction tests. Based on analysis of densification curve of gyratory compaction, influence rules of the contents of mineral aggregates of all sizes and asphalt on compaction characteristic of asphalt mixtures were obtained. SGC Tests show that, for the mixture with a bigger content of asphalt, its density increases faster, that there is an optimal amount of fine aggregates for optimal compaction and that an appropriate amount of mineral powder will improve workability of mixtures, but overmuch mineral powder will make mixtures dry and hard. Conclusions based on SGC tests can provide basis for how to adjust material composition for improving compaction performance of asphalt mixtures, and for the designed asphalt mixture, its compaction performance can be predicted through these conclusions, which also contributes to the choice of compaction schemes.

## 1. Introduction

Compaction characteristic of asphalt mixtures is often used to describe how easy or difficult it is to compact a mixture on a roadway. For the hot-mix asphalt mixtures, compaction has a great influence on its strength and durability. Good compaction can make asphalt mixtures acquire enough carrying capacity to meet the need of heavy traffic. However, compaction mechanism of asphalt concrete is very complicated and there are many influence factors. Any error in the process of compaction may do harm to the quality and field performance of the whole pavement. Influence factors of compaction performance of asphalt mixtures include many aspects; a lot of researches aim at temperature, rolling machines, and so on [1, 2]. Xiao and Wang [3] analyzed compaction performance of the multilevel interlocked dense type asphalt mixture through the lab rutting test. Stakston et al. [4] and Aho et al. [5] analyzed influence of aggregate shape on compaction characteristic of mixture through tests. Hussain and Timothy [6] proposed the concept of densification energy index through analysis of densification curve characteristic of gyratory compaction. Li et al. [7] researched influence of material composition (gradation, the content of asphalt) and compaction pressure on compaction characteristic through gyratory compaction tests, but it only compared the compaction characteristic of several gradations such as AC-13I, AK-13A, and Superpave gradations and did not analyze the influence rule of gradation variety on compaction characteristic systematically. Leiva and West [8] analyzed the basic compaction parameters (such as the compaction energy index, the slope of densification curve, and the number of gyrations required to reach 92% of the theoretical maximum density) and their relations through the lab gyratory compaction tests, providing a basis for analysis of influence of material composition (gradation, aggregate shape, binder grade, and so on) on compaction characteristic.

The internal material composition of asphalt mixture is the base of its all exterior characteristics; so compaction characteristic of asphalt mixture is mainly decided by its material composition. However, there are few deep and careful researches about influence rules of material composition on compaction characteristic. This paper will systematically analyze influence rules of material composition on compaction characteristic of asphalt mixtures through the SGC simulative compaction tests. This research aims at the surface layer asphalt mixture (13-type gradation); other types of mixtures will be discussed in the later other researches. In addition, this research only analyzes the influence rule and trend of compaction characteristic and does not judge if the compaction performance of some mixture is good or bad. The arrangement of this paper is as folows: Section 2 introduces the simulative compaction principle of Superpave gyratory compactor, Section 3 is the gyratory compaction tests and data analysis, and Section 4 is the conclusions.

## 2. Simulative Compaction Principle of Superpave Gyratory Compactor

Among many test instruments for researching compaction characteristic of asphalt mixtures, Superpave gyratory compactor can better simulate the compaction process of mixtures under rolling and vehicles. Through observation of height variety of test specimens in the lab gyratory compaction test, the densification characteristic of asphalt mixtures during construction and after traffic is open can be evaluated [9]. In this paper, Superpave gyratory compactor is used to analyze the influence of all kinds of material compositions on compaction characteristic through the lab tests, which cannot be done in the field tests. Strategic Highway Research Program (SHRP) researchers have several purposes in the development of lab compaction methods. The most important one is to compact the test specimen to the field density simulatively. The larger compaction equipment is needed to adapt to the mineral aggregates of big size. Compaction performance tests are needed to recognize the unstable mixtures and other compaction problems. SHRP researchers also considered the weight of equipment. Because the current compaction equipments did not meet these needs, so they developed the Superpave gyratory compactor (SGC).

SGC can satisfy the need of simulative compaction and its weight is also very light. The diameter of test specimens is 6 inches (150 mm) and can fit the mixtures composed of mineral aggregates of maximum size 50 mm (nominal maximum size 37.5 mm). Gyratory compaction molding can simulate the action on mixtures of construction machines and vehicles in the process of paving, rolling and traffic loading. Similar to the other mix design methods, the mixture is designed under a certain compaction level. In the Superpave design method, the compaction level is a function of the designed gyratory compaction number *N*_*des*⁡_ which is used to distinguish the difference of compaction effort. *N*_*des*⁡_ is a function of traffic level; the traffic level can be denoted by the designed ESAL (equivalent single axle load). The values of *N*_*des*⁡_ are listed in Table 1.

**Figure 1:**
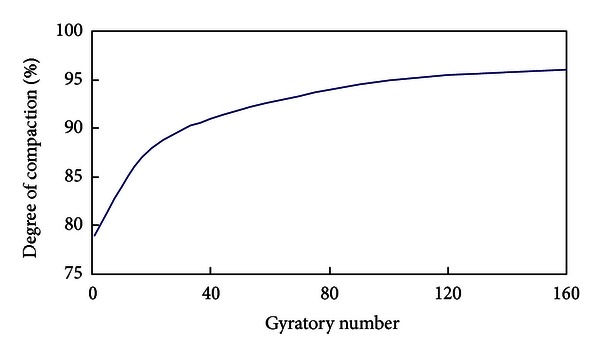
Relations of degree of compaction and gyratory number.

The initial gyratory number *N*_ini_ is equivalent to compaction effort of paving; the degree of compaction (ratio of compaction density to theoretical maximum density) needs to be under 89% to avoid soft asphalt mixtures. The designed gyratory number *N*_*des*⁡_ is equivalent to compaction effort of rolling and traffic loading until the mixture is stable; the degree of compaction is demanded to be equal to 96% to attain the designed air voids 4%. The maximum gyratory number *N*_max⁡_ is equivalent to compaction effort of lasting action of traffic loads. If the minimum air void is less than 2%, the mixture can be considered to have been destroyed; so the degree of compaction is demanded to be under 98% under the maximum gyratory number *N*_max⁡_ [11].  Figure 1 illustrated how the density of mixtures increases with gyratory number. In general, if the semilogarithmic coordinates are applied, the densification curve between *N*_ini_ and *N*_*des*⁡_ takes on the form of line, but the curve between *N*_*des*⁡_ and *N*_max⁡_ is not a perfect line [12]. So the whole densification curve can be divided into two sections; each section reflects the characteristic of different compaction phases.

This paper mainly analyzes the densification curve between gyratory number *N*_ini_ and *N*_*des*⁡_, namely, analyzing the compaction characteristic of mixtures from paving and rolling to attaining stability under traffic loads, also, namely, analyzing influence factors and rules of how easy or difficult the mixture is compacted to the designed air voids. For the densification curve between *N*_*des*⁡_ and *N*_max⁡_ (viz., compaction process from the stability phase to the minimum air voids), the main problem is to judge if mixtures will be destroyed (viz., air voids less than 2%), which may be discussed in the later other researches and is not studied in this paper. This paper only researches compaction characteristic of the first phase. Because the densification curve between *N*_ini_ and *N*_*des*⁡_ on the semilogarithmic coordinates takes on the form of line; so this research will mainly use the semilogarithm coordinates for simplifying analysis.

## 3. Gyratory Compaction Tests and Data Analysis

Gyratory number 125 under a pressure of 600 kPa mainly analyzes the densification curve between gyratory number *N*_ini_ and *N*_*des*⁡_, namely, studying compaction characteristic from paving and rolling to attaining stability under traffic loads, also, namely, analyzing how easy or difficult the mixture is compacted to the designed air voids. Here, according to Table 1,  *N*_ini_ takes 8; *N*_*des*⁡_ takes 100.

### 3.1. Gyratory Compaction Tests

This research aims at asphalt mixtures of 13-type gradation. According to analysis needs, the content of mineral aggregates and asphalt of all test schemes is made in Table 2. 3.5% and 4.5% for the content of the bitumen are because they stood for two typical level of bitumen content of general mixtures. Mineral aggregates are basalts; mineral powder is limestone (size less than 0.15 mm). Heavy-traffic asphalt AH-90 is used in the tests. The compaction temperature for asphalt mixtures is about 160°C.

Each test scheme molds 3 test specimens; the mix volumetric properties (average values of 3 specimens) are measured after gyratory number 125 (Table 3).

Record the specimen height of each gyratory number in the compaction process (SGC automatically records), calculate the specimen bulk volume density of each gyratory number according to (1), and calculate the degree of compaction according to (2).

The bulk volume density *ρ* of the specimen height *h* is approximately

(1) $$\begin{array}{c} \rho =\frac{\rho_{b}}{m_{0}/\left(\pi r^{2}h_{t}\right)}\left(\frac{m_{0}}{\pi r^{2}h}\right). \end{array}$$

In (1), *ρ*_*b*_ is the specimen bulk volume density under gyratory number 125, *m*_0_ is the dry weight of specimens, *r* is the specimen radius (7.5 cm), and *h*_*t*_ is the specimen height of gyratory number 125.

Degree of compaction *γ* is

(2) $$\begin{array}{c} \gamma =\frac{\rho }{G_{\text{mm}}}\times 100\%. \end{array}$$

In (2), *G*_mm_ is the theoretical maximum density of asphalt mixtures.

Then for each test scheme, average the degree of compaction of 3 specimens (Table 4). Densification curve of each test scheme can also be drawn.

### 3.2. Data Analysis

Data analysis indexes include the slope of densification curve between *N*_ini_ and *N*_*des*⁡_, the degree of compaction of gyratory number 8, the degree of compaction of gyratory number 100 and the number of gyrations required to reach 92% of the theoretical maximum density (*G*_mm_).

On the semilogarithmic coordinates, curve slope reflects the increasing speed of mix density in the compaction process. The degree of compaction of gyratory number 8 can show if the mixture is a soft asphalt mixture. The degree of compaction of gyratory number 100 can show the air void of the mixture when it attains stability under traffic loads. Because many specifications [10] all demand pavements to be compacted to a density of 92%  *G*_mm_, the number of gyrations required to reach 92%  *G*_mm_ can be found in the densification curve; if this number is big, construction compaction is difficult.

The following comparison data are from SGC tests (see Table 4).

#### 3.2.1. Compare Test Schemes (3) and (8) and Analyze Influence of Variety of Binder Content on Compaction Characteristic

**Figure 2:**
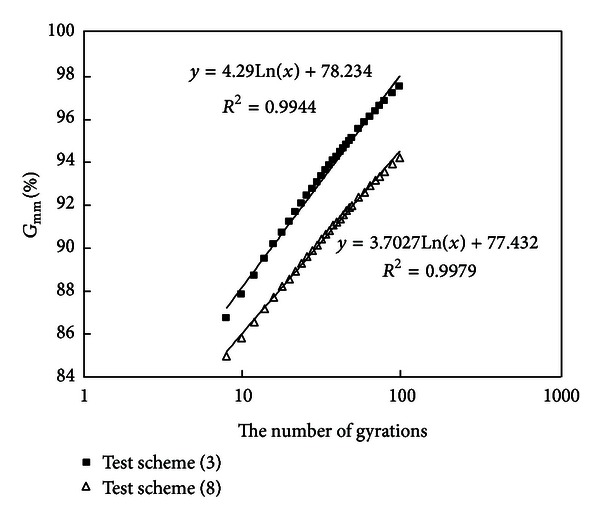
Densification curves of schemes (3) and (8).

Densification curves of schemes (3) and (8) are drawn in Figure 2.

It is known from Table 2 that the gradations of test schemes (3) and (8) are the same, but binder content of test scheme (3) is 1% more than that of test scheme (8).

*Slope Comparison*. The slope of densification curve of scheme (3) is a little higher than that of scheme (8); so a higher binder content will make density-increasing speed become faster.

*Density of Gyratory Number 8*. Mixture (3) with a higher content of asphalt has a higher density (86.7%  *G*_mm_, Table 4); mixture (8) with a low content of asphalt has a small density (84.9%  *G*_mm_). There is a difference of 1.8%  *G*_mm_ between mixtures (3) and (8), which shows more of asphalt improved initial workability of mixtures.

*Density of Gyratory Number 100*. Mixture (3) with a higher content of asphalt has a higher density (97.5%  *G*_mm_); mixture (8) with a low content of asphalt has a small density (94.2%  *G*_mm_). There is a difference of 3.3%  *G*_mm_ between mixtures (3) and (8).

*The Number of Gyrations Required to Reach 92%* *G*_mm_. It is known from Figure 2 that gyratory number of scheme (3) (higher binder content) is smaller, which shows increasing binder content will facilitate construction compaction. 

#### 3.2.2. Analyze the Results of Test Schemes (1), (2), (3), and (4) and Study the Influence of Ratio of Coarse Aggregates to Fine Aggregates on Compaction Characteristic

**Figure 3:**
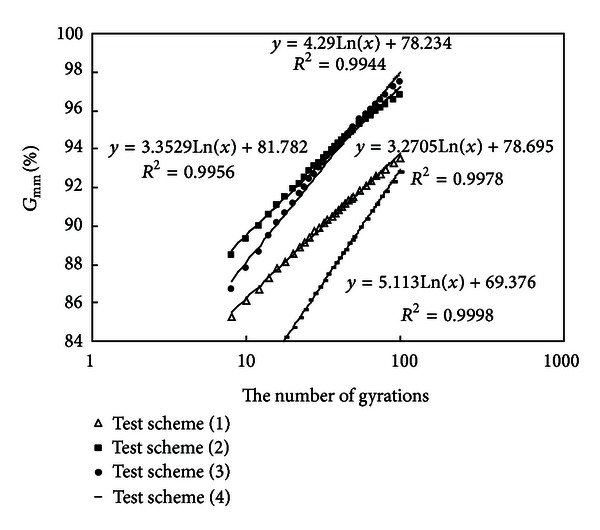
Densification curves of schemes (1), (2), (3), and (4).

Densification curves of mixtures (1), (2), (3) and (4) are drawn in Figure 3.

It is known from Table 2 that schemes (1), (2), (3), and (4) have an equal binder content and an equal mineral powder content, but the content of coarse aggregates increases gradually and that of fine aggregates decreases gradually.

Compare mixtures (1) with (2); the content of fine aggregates less than 4.75 mm decreases from 60% to 42% while coarse aggregates increases from 30% to 48%.

*Slope Comparison*. Densification curves of schemes (1) and (2) are nearly parallel; so their density-increasing speeds are nearly the same.

*Density of Gyratory Number 8.* The density of mixture (1) is 85.3%  *G*_mm_; the density of mixture (2) is 88.5%  *G*_mm_. There is a difference of 3.2%  *G*_mm_ between mixtures (1) and (2); overmany fine aggregates will make the initial air voids become large.

*Density of Gyratory Number 100.* The density of mixture (1) is 93.5%  *G*_mm_; the density of mixture (2) is 96.8%  *G*_mm_. There is a difference of 3.3%  *G*_mm_ between mixtures (1) and (2); overmany fine aggregates will also make the final air voids become large.

*The Number of Gyrations Required to Reach 92%* *G*_mm_. It is known from Figure 3 that gyratory number of scheme (2) is much smaller than that of scheme (1), which shows that overmany fine aggregates will make specific surface area become large and make oil film become thin, thus making compaction become more difficult.

Compare mixtures (2) with (3); the content of fine aggregates less than 4.75 mm decreases from 42% to 24% while coarse aggregates increases from 48% to 66%.

*Slope Comparison.* Slope of densification curve of scheme (3) is higher than that of test scheme (2), which shows that when the content of coarse aggregates adds to a certain amount, the density-increasing speed becomes faster.

*Density of Gyratory Number 8*. The density of mixture (3) is 86.7%  *G*_mm_; the density of mixture (2) is 88.5%  *G*_mm_. There is a difference of 3.2%  *G*_mm_ between mixtures (2) and (3). When the content of coarse aggregates adds to a certain amount, the initial air voids start to become large. 

*Density of Gyratory Number 100*. The density of mixture (3) is 97.5%  *G*_mm_; the density of mixture (2) is 96.8%  *G*_mm_. There is a difference of 0.7%  *G*_mm_ between mixtures (2) and (3). The final air voids continue to decrease. 

*The Number of Gyrations Required to Reach 92%* *G*_mm_. It is known from Figure 3 that gyratory number of scheme (3) is a little more than that of scheme (2), which shows that compaction characteristic of mixtures will change and construction compaction become difficult when the content of fine aggregates decreases to a certain amount.

Compare mixtures (3) with (4); the content of fine aggregates less than 4.75 mm decreases from 24% to 6% while coarse aggregates increases from 66% to 84%.

*Slope Comparison.* Slope of densification curve of scheme (4) is higher. The content of coarse aggregates continues to add; the density-increasing speed continues to become greater.

*Density of Gyratory Number 8*. The density of mixture (4) is 80.0%  *G*_mm_; the density of mixture (3) is 86.7%  *G*_mm_. There is a difference of 6.7%  *G*_mm_ between mixtures (3) and (4). When coarse aggregates continue to increase, the initial density of mixtures continues to fall (air voids become larger). 

*Density of Gyratory Number 100*. The density of mixture (4) is 92.7%  *G*_mm_; the density of mixture (3) is 97.5%  *G*_mm_. There is a difference of 4.8%  *G*_mm_ between mixtures (3) and (4). When coarse aggregates continue to increase, the final density of mixtures starts to fall (air voids become large) owing to lack of enough fine aggregates filled in coarse ones.

*The Number of Gyrations Required to Reach 92% G*_mm_. It is known from Figure 3 that gyratory number of scheme (4) is much more than that of scheme (3), which shows that overmany coarse aggregates and oversmall fine aggregates will make construction compaction become very difficult because a strong interlocking strength is formed between coarse aggregates and so the strong inner frictional resistance needs to be conquered in the compaction process.

**Figure 4:**
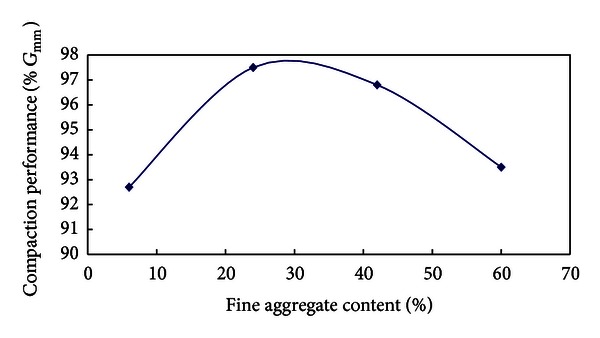
Variety rule of compaction performance with fine aggregate content.

From the above analysis, variety rules of compaction performance with the content of fine aggregates are depicted in Figure 4.

#### 3.2.3. Compare the Results of Test Schemes of (5), (10), and (11)

**Figure 5:**
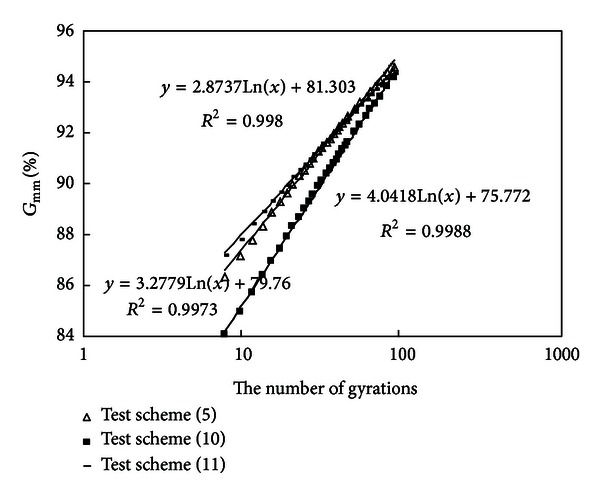
Densification curves of schemes (5), (10), and (11).

Densification curves of schemes (5), (10), and (11) are drawn in Figure 5.

Compare mixtures (10) with (11); they have the same contents of coarse aggregates above 4.75 mm, the same contents of mineral powder, and the same contents of asphalt, but mixture (10) has more of thicker fine aggregates while mixture (11) has more of thinner fine aggregates.

*Slope Comparison*. Slope of densification curve of test scheme (10) is higher; the mixture having more of thicker fine aggregates has a higher density-increasing speed.

*Density of Gyratory Number 8*. The density of mixture (10) is 84.0%  *G*_mm_; the density of mixture (11) is 87.1%  *G*_mm_. The density of mixture (10) is 3.1%  *G*_mm_ lower than that of mixture (11); the mixture having more of thicker fine aggregates has a low initial density and a high air void. 

*Density of Gyratory Number 100.* Being nearly the same, the density of mixture (10) is 94.1%  *G*_mm_; and that of mixture (11) 94.3%  *G*_mm_.

*The Number of Gyrations Required to Reach 92%* *G*_mm_. It is known from Figure 5 that gyratory number of scheme (10) is more, which is because the thicker fine aggregates have a stronger inner frictional resistance.

Compare mixtures (5) with (11); they have the same contents of coarse aggregates above 4.75 mm and the same contents of asphalt, but mixture (5) has more of thicker fine aggregates.

*Slope Comparison*. Slope of densification curve of test scheme (5) is a little higher.

*Density of Gyratory Number 8*. The initial density of mixture (5) is a little lower than that of mixture (11).

*Density of Gyratory Number 100*. There is no big difference between the two mixtures.

*The Number of Gyrations Required to Reach 92%* *G*_mm_. There is no big difference between the two mixtures.

Compare mixtures (5) with (10); they have the same contents of coarse aggregates and the same contents of asphalt; mixture (10) has more of thicker fine aggregates and mineral powder but less of thinner fine aggregates.

*Slope Comparison*. Slope of densification curve of test scheme (10) is a little higher.

*Density of Gyratory Number 8*. The density of mixture (5) is 86.3%  *G*_mm_; and that of mixture (10) 84.0%  *G*_mm_. The mixture having more of thicker fine aggregates has a higher density-increasing speed. 

*Density of Gyratory Number 100*. There is no big difference between the two mixtures.

*The Number of Gyrations Required to Reach 92% G*_mm_. Gyratory number of scheme (10) is more; this is because scheme (10) has a big amount of thicker fine aggregates which produce a strong inner frictional resistance; so construction compaction performance of mixture (10) is worse than that of mixture (5); this also shows that the effect of thicker fine aggregates on compaction performance is prominent.

#### 3.2.4. Compare the Results of Schemes (3) and (9)

**Figure 6:**
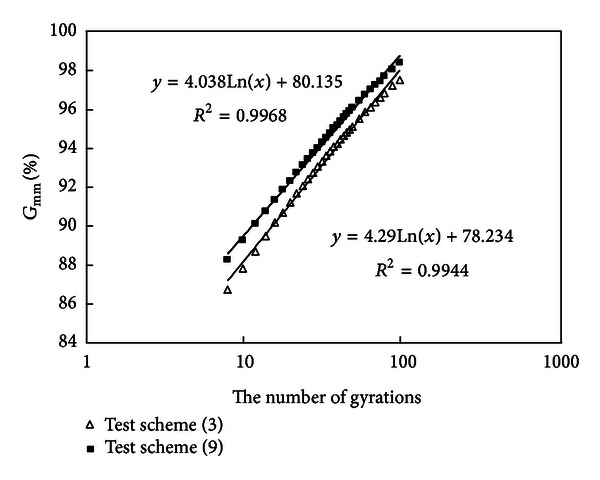
Densification curves of schemes (3) and (9).

Densification curves of schemes (3) and (9) are drawn in Figure 6.

Mixtures (3) and (9) have the same amount of fine aggregates, mineral powder, and asphalt, but mixture (9) has more of thicker coarse aggregates. Their final ail voids have no big difference, but the initial air void of mixture (9) is a little lower, which shows the proportion of each size section among coarse aggregates above 4.75 mm has no obvious influence on compaction characteristic, may be more of thicker coarse aggregates will contribute to compaction; this is probably because there are less contact points between coarse aggregate particles.

#### 3.2.5. Compare the Results of Schemes (7) and (8)

**Figure 7:**
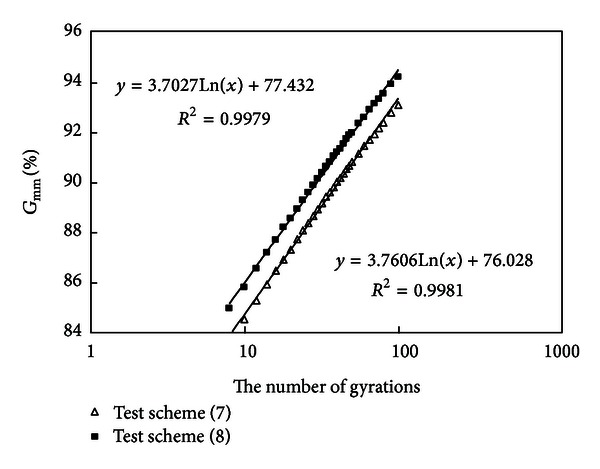
Densification curves of schemes (7) and (8).

Densification curves of schemes (7) and (8) are drawn in Figure 7.

Mixtures (7) and (8) have the same amount of coarse aggregates and asphalt, but mixture (7) has a less amount of mineral powder. It is known from Figure 7 that the slopes of two curves are nearly equal; namely, their density-increasing speeds are nearly equal. But mixture (8) is more easy to compact (needing less of gyrations for attaining a density of 92%  *G*_mm_) than mixture (7) because the more mineral powder caused a smaller air void, which also shows that an appropriate amount of mineral powder will contribute to compaction.

#### 3.2.6. Compare the Results of Schemes (2) and (5)

**Figure 8:**
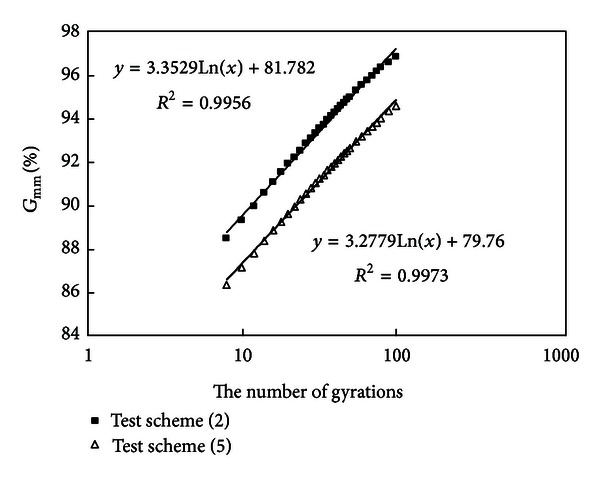
Densification curves of schemes (2) and (5).

Densification curves of schemes (2) and (5) are drawn in Figure 8.

Similarly to analysis of (5), compare mixture (2) with (5), they have the same amount of coarse aggregates and asphalt, but mixture (2) has a higher amount of mineral powder (10%) and mixture (5) has a lower amount of mineral powder (4%). It is known from Figure 8 that the slopes of two curves are nearly equal, but mixture (2) has a smaller air void and mixture (2) is more easy to compact than mixture (5) because it needs less of gyrations for attaining a density of 92%  *G*_mm_, which shows that an appropriate amount of mineral powder will improve workability of mixtures and contribute to field construction compaction.

#### 3.2.7. Compare the Results of Schemes (3) and (6)

**Figure 9:**
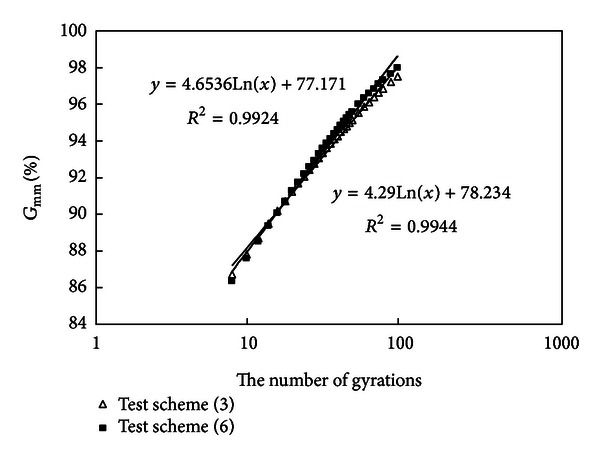
Densification curves of schemes (3) and (6).

Densification curves of schemes (3) and (6) are drawn in Figure 9.

Mixtures (3) and (6) have the same amount of asphalt and the contents of coarse aggregates are also nearly equal, but mixture (6) has a higher amount of mineral powder (16%). It is known from Figure 9 that two curves almost overlap and scheme (6) has a little higher slope and a little lower final air void. Mineral powder continuing to add after it has added to a certain amount will not do good to compaction any longer; this is because overmuch mineral powder will make mixtures dry and do harm to field compaction.

#### 3.2.8. Compare the Results of Schemes (3) and (7)

**Figure 10:**
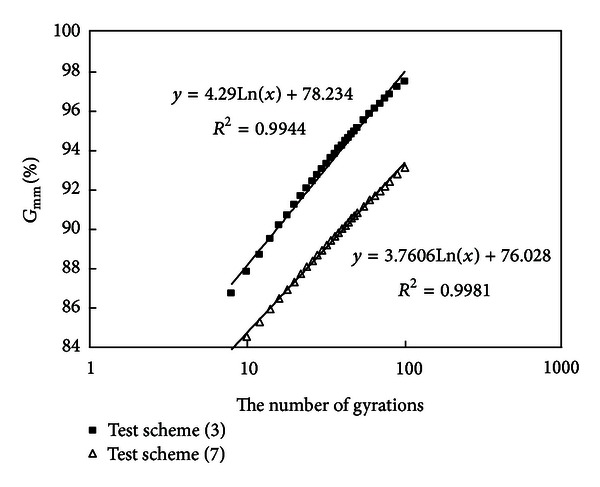
Densification curves of schemes (3) and (7).

Densification curves of schemes (3) and (7) are drawn in Figure 10.

Mixtures (3) and (7) have the same amount of coarse aggregates, but mixture (3) has a higher amount of mineral powder and a higher amount of asphalt. It is known from Figure 10 that mixture (3) has a smaller air void and the slope of densification curve of scheme (3) is a little higher. The density-increasing speed of mixture (3) is faster and the number of gyrations required to reach 92%  *G*_mm_ is also much smaller than that of scheme (7).

#### 3.2.9. Compare the Results of Schemes (5) and (6)

**Figure 11:**
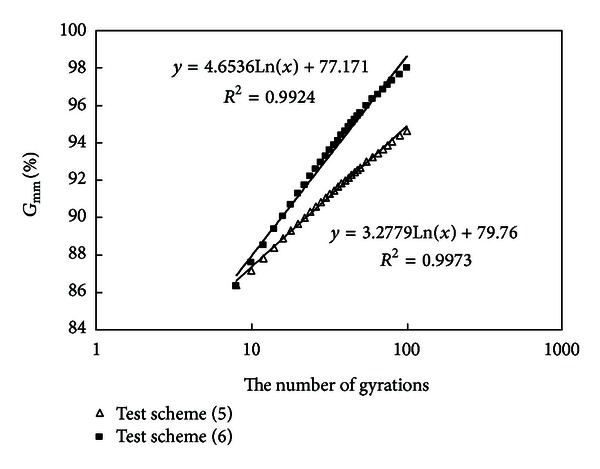
Densification curves of schemes (5) and (6).

Densification curves of schemes (5) and (6) are drawn in Figure 11.

Mixtures (5) and (6) have the same amount of asphalt; mixture (6) has a higher amount of coarse aggregates above 4.75 mm (69%), a higher amount of mineral powder (16%), and a lower amount of fine aggregates (15%); comparatively, mixture (5) has a higher amount of fine aggregates (48%) and a lower amount of mineral powder (only 4%). It is known from Figure 11 that the final air void of mixture (6) is very small; its curve slope is big, so the density-increasing speed is fast; the number of gyrations required to reach 92%  *G*_mm_ is small, it is more easy to compact than mixture (5) but has an oversmall final air void; mixture (5) has overmany fine aggregates but lacks enough mineral powder; so it is more difficult to compact and the final air void is big.

## 4. Conclusions

The slope of densification curve cannot be used as the only criterion for judging whether compaction is difficult or easy; otherwise, the inaccurate conclusion that more coarse aggregates make compaction easier may be drawn. The mixture having more of asphalt has a faster density-increasing speed; it is more easy to compact and has a smaller air void when their gradations are the same. 

Test schemes (1), (2), (3), and (4) have the same amount of asphalt and mineral powder, but the content of coarse aggregates increases gradually and that of fine aggregates decreases gradually; the variety rule of compaction characteristic with the content of fine aggregates is depicted in Figure 4. It can be seen from Figure 4 that when the content of fine aggregates is small and that of coarse aggregates is large the mixture is difficult to compact; with the gradual increase of fine aggregates compaction performance of mixtures improves gradually; but with the continual increase of fine aggregates compaction performance of mixtures starts to fall. So there is an optimal amount of fine aggregates.

When the contents of coarse aggregates, mineral powder, and asphalt are all kept constant, the thicker ones in fine aggregates has a prominent effect on compaction performance; more of thicker ones will make compaction more difficult. When the contents of fine aggregates, mineral powder, and asphalt are all kept constant, the proportion of each size section among coarse aggregates above 4.75 mm has no obvious influence on compaction characteristic; maybe more of thicker coarse aggregates will contribute to compaction; this is probably because there are less of contact points between coarse aggregate particles. When the contents of coarse aggregates and asphalt are kept fixed, the air void of the mixture with more mineral powder is smaller and more easy to compact, which shows that an appropriate amount of mineral powder will improve workability of mixtures and contribute to field compaction.

Mineral powder continuing to add after it has added to a certain amount will not do good to compaction any longer; this is because overmuch mineral powder will make mixtures dry and hard and do harm to field construction compaction. When the content of coarse aggregates is kept constant, the mixture having more of mineral powder and asphalt has a smaller air void and its density-increasing speed is faster. The mixture having more of coarse aggregates and mineral powder but less of fine aggregates has a smaller final air void, and its density-increasing speed is fast; although it is more easy to compact, its final air void will be too small. The mixture having overmany fine aggregates but lacking enough mineral powder is more difficult to compact and the final air void is usually large.

## Figures and Tables

**Table 1:** Superpave gyratory compaction parameters (AASHTO PP28-00) [10].

Designed ESAL/10^6^	Compaction parameters			Typical road applications
	*N*_ini_	*N*_*des*⁡_	*N*_max⁡_	
<0.3	6	50	75	Light-traffic roads, such as local roads and county roads
0.3~<3	7	75	115	Distributed roads, entrance roads into blocks, city roads of medium traffic, and parts of county roads
3~<30	8	100	160	Two-lane roads, multilane roads, entrance roads into cities, city roads of medium or heavy traffic, state roads, and country roads
≥30	9	125	205	Cross-state roads, roads of heavy traffic, and truck-exclusive roads

**Table 2:** Content of mineral aggregates and asphalt of all test schemes.

Test schemes	Percentage content of mineral aggregates of each size section (%)									Asphalt-aggregate ratio (%)
	13.2~16 mm	9.5~13.2 mm	4.75~9.5 mm	2.36~4.75 mm	1.18~2.36 mm	0.6~1.18 mm	0.3~0.6 mm	0.15~0.3 mm	Mineral powder	
(1)	5	10	15	12	12	12	12	12	10	4.5
(2)	8	16	24	8	8	8	9	9	10	4.5
(3)	11	22	33	4	5	5	5	5	10	4.5
(4)	14	28	42	1	1	1	1	2	10	4.5
(5)	8	16	24	9	9	10	10	10	4	4.5
(6)	12	23	34	3	3	3	3	3	16	4.5
(7)	11	22	33	5	6	6	6	6	5	3.5
(8)	11	22	33	4	5	5	5	5	10	3.5
(9)	22	33	11	4	5	5	5	5	10	4.5
(10)	8	16	24	10	16	16	0	0	10	4.5
(11)	8	16	24	4	0	4	14	20	10	4.5

**Table 3:** Mix volumetric properties of all test schemes.

Test schemes	Bulk volume density (g/cm^3^)	Theoretical maximum density (g/cm^3^)	Air voids (%)	Percent voids in coarse mineral aggregate (%)
(1)	2.401	2.553	5.93	74.8
(2)	2.486	2.555	2.69	58.3
(3)	2.51	2.558	1.89	42.1
(4)	2.401	2.560	6.24	29.5
(5)	2.43	2.554	4.86	59.2
(6)	2.522	2.559	1.45	39.1
(7)	2.432	2.595	6.25	43.3
(8)	2.461	2.595	5.17	42.7
(9)	2.527	2.555	1.12	41.6
(10)	2.432	2.565	5.17	59.2
(11)	2.412	2.542	5.14	59.5

**Table 4:** Degree of compaction of each gyratory number (%) (parts of data).

Gyratory number	Test schemes										
	(1)	(2)	(3)	(4)	(5)	(6)	(7)	(8)	(9)	(10)	(11)
8	85.3	88.5	86.7	80.0	86.3	86.3	83.6	84.9	88.2	84.0	87.1
12	86.7	89.9	88.7	82.0	87.8	88.5	85.3	86.5	90.1	85.7	88.4
20	88.6	91.9	91.2	84.7	89.6	91.2	87.3	88.5	92.3	87.9	89.9
30	89.9	93.3	93.0	86.8	91.0	93.2	88.9	90.1	94.0	89.6	91.1
40	90.9	94.3	94.2	88.3	91.9	94.5	90.0	91.2	95.1	90.8	92.0
50	91.5	95.0	95.1	89.4	92.6	95.5	90.8	92.0	96.0	91.6	92.6
60	92.1	95.5	95.8	90.4	93.2	96.3	91.5	92.6	96.7	92.3	93.1
70	92.6	96.0	96.3	91.1	93.6	96.8	91.9	93.1	97.2	92.9	93.5
80	93.0	96.3	96.8	91.8	94.0	97.3	92.4	93.5	97.7	93.4	93.8
90	93.3	96.6	97.2	92.3	94.3	97.6	92.8	93.9	98.0	93.8	94.1
100	93.5	96.8	97.5	92.7	94.6	97.9	93.1	94.2	98.4	94.1	94.3
110	93.8	97.1	97.8	93.2	94.8	98.2	93.3	94.5	98.5	94.4	94.6
125	94.1	97.3	98.1	93.8	95.1	98.6	93.7	94.8	98.9	94.8	94.9
